# A Comparison of the Malnutrition Universal Screening Tool (MUST) and the Mini Nutritional Assessment-Short Form (MNA-SF) Tool for Older Patients Undergoing General Surgery

**DOI:** 10.3390/jcm10245860

**Published:** 2021-12-14

**Authors:** Stamatios Kokkinakis, Maria Venianaki, Georgia Petra, Alexandros Chrysos, Emmanuel Chrysos, Konstantinos Lasithiotakis

**Affiliations:** 1 Department of General Surgery, University General Hospital of Heraklion, 71110 Heraklion, Greece; stamatioskokkinakis@gmail.com (S.K.); mb12.med@gmail.com (M.V.); geopetra94@gmail.com (G.P.); chrysose@uoc.gr (E.C.); 2 Department of General, Visceral and Transplantation Surgery, University Clinic RWTH Aachen, Pauwelsstrasse 30, 52074 Aachen, Germany; alxchrysos@gmail.com

**Keywords:** geriatric surgery, malnutrition, mini nutritional assessment short-form, malnutrition universal screening tool

## Abstract

The optimal malnutrition screening tool in geriatric surgery has yet to be determined. Herein, we compare two main tools in older patients undergoing general surgery operations. Older patients (>65 years old) who underwent general surgery operations between 2012 and 2017 in a tertiary centre were included. The Malnutrition Universal Screening Tool (MUST) and the Mini Nutritional Assessment Short Form (MNA-SF) were used for nutritional risk assessment. Preoperative variables as well as postoperative outcomes were recorded prospectively. Agreement between tools was determined with the weighted kappa (κ) statistic. Multiple regression analysis was used to assess the association of the screening tools with postoperative outcomes. A total of 302 patients (median age 74 years, range: 65–92) were included. A similar number of patients were classified as medium/high risk for malnutrition with the MNA-SF and MUST (26% vs. 36%, *p* = 0.126). Agreement between the two tools was moderate (weighted κ: 0.474; 95%CI: 0.381–0.568). In the multivariate analysis, MNA-SF was associated significantly with postoperative mortality (*p* = 0.038) and with postoperative length of stay (*p* = 0.001). MUST was associated with postoperative length of stay (*p* = 0.048). The MNA-SF seems to be more consistently associated with postoperative outcomes in elderly patients undergoing general surgery compared with the MUST tool.

## 1. Introduction

Elderly patients undergoing general surgical procedures are at higher risk for postoperative complications compared with their younger counterparts. Surgical stress leads to a catabolic state and it is known that muscle recovery following disuse in elderly patients is slower and does not reach the extent observed in younger individuals [1]. There are several reports which document the impact of malnutrition in postoperative outcomes in the elderly [2,3,4]. Patients at nutritional risk have a higher rate of postoperative complications, higher mortality and a longer hospital stay [5]. The prevalence of malnutrition detected during the preoperative assessment of surgical patients is consistently high in recent studies [6,7]. The recent European Society for Clinical Nutrition and Metabolism (ESPEN) recommendations on perioperative nutrition suggest that body mass index (BMI) along with the presence of unintentional weight loss, anorexia, reduced oral intake and severity of comorbid conditions are the main criteria suitable for identifying patients at high nutritional risk [8]. However, no clear recommendation is made on the preferred screening method for malnutrition. Single parameter based nutritional risk assessment considering for example serum albumin, body mass index etc. have been evaluated in the past but they were not precise in identifying patients at risk for malnutrition [8]. Therefore, validated nutritional screening tools were developed using combinations of various criteria as a more accurate method for assessing the risk for malnutrition. Despite the availability of a range of malnutrition screening tools there is no solid evidence regarding which one should be preferably used in the preoperative setting of older patients. Expert consensus recommends the Malnutrition Universal Screening Tool (MUST) for use in the community and the Mini Nutritional Assessment Short Form (MNA-SF) in the elderly [8]. Therefore, the aim of this study was to compare MUST and the MNA-SF in elderly patients undergoing general surgery, with regards to their association with postoperative outcomes.

## 2. Materials and Methods

### 2.1. Study Population

Between 2012 and 2017, elderly patients (>65 years old), who were scheduled to undergo general surgery procedures in our department were assessed in the context of a comprehensive geriatric assessment protocol. Patients unable to undergo nutritional assessment (i.e., due to poor physical or mental status) and/or provide written consent were excluded from the study. Written informed consent was provided by all patients and the study protocol was approved by the Institutional Scientific and Ethical Committee. All patients included in the study underwent physical examination and were interviewed by a senior surgical trainee. In patients with cognitive impairment, necessary information was gathered or confirmed by their closest relative or caregiver.

### 2.2. Perioperative Data

For nutritional assessment, the MUST and the MNA-SF were used. MUST was developed by the British Association for Parenteral and Enteral Nutrition (BAPEN) in order to identify adults at risk of malnutrition [9]. A total of 3 main parameters are used in MUST: patient BMI, unintentional weight loss in the past 3–6 months, and acute disease effect, implying a patient that is acutely ill and there has been or is likely to be no nutritional intake for >5 days. The first 2 parameters receive 0, 1 or 2 points each, and the last parameter receives 2 points in case of positivity. A total score of 0, 1 and ≥2 denotes low, medium and high risk for malnutrition, respectively. The MNA-SF has been validated as a valuable screening tool in the elderly population [10,11]. A total of 6 parameters are assessed in the MNA-SF tool: food intake, unintentional weight loss, mobility, psychological stress or acute disease, neuropsychological problems, and BMI or calf circumference. According to the MNA-SF, patients are classified as having normal nutritional status (12–14 points), being at risk of malnutrition (8–11 points) or being malnourished (0–7 points). Self-maintaining and instrumental activities were assessed using the Katz basic activities of daily life index (ADL) [12], which includes 6 items that assess basic self-care activities such as bathing, dressing, clothing, toileting, feeding, transferring and continence. A score of 0–2 denotes a dependent patient, 3–4 an intermediate and 5–6 an independent patient. Comorbidity was assessed using the Charlson comorbidity index (CCI) [13]. The American Society of Anesthesiologists (ASA) classification is a physical status classification system which consists of 5 categories of increasing severity [14]. Very briefly, ASA Class I denotes a completely healthy fit patient. ASA II and III denote a patient with mild systemic and severe systemic disease that is not incapacitating, respectively. ASA IV refers to a patient with an incapacitating disease that is a constant threat to life and Class V a moribund patient. The magnitude of the operations was assessed using the Physiological and operative severity score for the enumeration of mortality and Morbidity (POSSUM) categories (minor, intermediate, major, major plus) [15] and the site of operation was grouped in 6 categories (hernia, upper gastrointestinal tract (GI), hepatobiliary/pancreatic (HPB), cholecystectomy, lower gastrointestinal tract (GI), soft tissue/other).

Postoperative complications and length of stay (LOS) were prospectively registered in the database. Complications were graded according to the classification proposed by Dindo et al. [16], divided in 5 grades. Grade I included any deviation from the normal postoperative course without the need for pharmacological treatment, or surgical, endoscopic and radiological interventions. Grade II require pharmacologic treatment, while Grade III complications require either surgical or endoscopic/radiological intervention. Grade IV includes life-threatening complications requiring IC/ICU management and Grade V is the death of a patient. Grade III-V are considered major complications.

### 2.3. Statistical Analysis

Categorical variables were presented as numbers (percentage) and continuous variables were presented as mean ± standard deviation (SD) if they were normally distributed or as median (IQR, interquartile range) if they did not follow the normal distribution. For the comparison of distribution of continuous variables, parametric or non-parametric tests were used. Relative risks were estimated using exposure odds ratios (ORs) and the corresponding 95% confidence intervals (CIs) from cross tabulation. Adjustment of the ORs for the effect of confounding factors was performed with multivariate logistic regression analysis. As postoperative LOS was not normally distributed, negative binomial regression analysis was performed to determine associated factors. All *p* values were two sided and the significance level was chosen to be 0.05. A weighted kappa-statistic was used to determine the degree of agreement between the two tools [17]. All calculations were performed with the Statistical Package for Social Sciences (SPSS) ver. 26.0 (SPSS Inc., Chicago, IL, USA).

## 3. Results

A total of 302 patients were included in the final analysis. The demographics and perioperative variables are shown in Table 1. The median (IQR) age of the population was 74 (10) years. A total of 26% of patients were deemed to be at risk of malnutrition or malnourished according to the MNA-SF tool, while 36% were classified as medium/high risk according to the MUST tool (*p* = 0.126). The degree of agreement between the two tools was deemed to be moderate according to the weighted kappa-statistic (weighted κ: 0.474; 95%CI: 0.381–0.568).

Table 2 shows the association between the two malnutrition risk assessment tools and the occurrence of postoperative complications, deaths and length of stay. In the univariate analysis, overall complications (*p* = 0.014), serious complications (*p* = 0.011), postoperative mortality (*p* = 0.029) and length of stay (*p* < 0.001) were all significantly higher in patients considered “at risk” of malnutrition according to the MNA-SF tool. Patients at “high risk” according to the MUST tool had a higher rate of overall complications (*p* = 0.026) and longer length of stay (*p* < 0.001). When “at risk” and “malnourished” for MNA-SF, and “medium risk” with “high risk” for MUST were grouped and examined together, “at risk/malnourished” MNA-SF scores were significantly associated with overall complications (*p* = 0.02), serious complications (*p* = 0.03), postoperative mortality (*p* = 0.018) and length of stay (*p* < 0.001). “Medium risk/high risk” MUST scores were significantly associated with overall postoperative complications (*p* = 0.02) and length of stay (*p* < 0.001). The distribution of malnutrition assessed by MNA-SF or MUST risk did not differ significantly between age groups. There was a statistically significant association of ASA class with malnutrition risk assessed by MUST but not by MNA-SF tool (Supplement Table S1). The associations of malnutrition risk with other preoperative variables is presented in Supplement Table S2. Patients undergoing HPB and lower GI surgery have significant higher rates of complications and are at higher risk for malnutrition assessed by both screening tools (*p* < 0.05) (Supplement Tables S3 and S4). The association of malnutrition risk with postoperative outcomes in patients undergoing HPB/GI operations versus other site of operation is presented in Supplement Table S5. Multivariate regression analysis is shown in Table 3. Patients “at risk” with the MNA-SF tool had significantly higher postoperative mortality and length of stay, while MUST “medium risk” patients had only longer postoperative length of stay. The detailed multivariate analysis is presented in Tables S6–S21 of Supplement file.

## 4. Discussion

This is the first study to compare the MNA-SF and the MUST tools in elderly patients undergoing general surgical procedures, focusing on their association with postoperative outcomes. The prevalence of patients judged to be at risk of malnutrition or malnourished in our study was 26% according to the MNA-SF, while 36% of our participants were judged to be at medium/high risk according to the MUST tool, and this high prevalence has already been noted in the literature. Zhou et al. compared the MNA-SF and the Nutrition Risk Screening 2002 (NRS 2002) in elderly surgical patients. Both tools detected a high prevalence of malnutrition among study participants, while agreement between the two tools was moderate. Patients with gastrointestinal disease were found to be malnourished in a greater extent with the MNA-SF [6]. In a Chinese study comparing the diagnostic value of three screening tools in geriatric patients with gastrointestinal cancer, the prevalence of patients at risk of malnutrition was high, and MUST identified a greater number of malnourished patients compared to the NRS 2002 and the MNA-SF [18]. It must be noted, however, that the authors used the new ESPEN guidelines as ‘gold standard’ for the diagnosis of malnutrition.

Comparisons between malnutrition screening tools with regard to complications have been made in different clinical settings. Lomivorotov et al. compared four malnutrition screening tools in cardiac surgery patients, and concluded that the MNA-SF detected a higher number of elderly patients at risk of malnutrition or malnourished, but the MUST tool was significantly associated with postoperative complications [19]. However, the authors point out the low levels of sensitivity in all screening tools, and the fact that their differences were ‘too small to have any clinical importance’. In a study including both medical and surgical patients, the NRS 2002 was deemed to be more valid than the MNA-SF in predicting complications and the agreement between the two tools using the kappa index was moderate, similar to our study [20]. Regarding the association of screening tools and patient survival, Charlton et al. investigated the long-term outcomes of hospitalised geriatric patients, and concluded that patients at risk/malnourished with the MNA-SF had lower survival curves at 18 months, and were more likely to be discharged to a different level of care than their well-nourished counterparts [21]. In a series of 101 geriatric cardiac surgery patients, unintentional weight loss prior to surgery was an independent predictor of 1-year mortality, while the MNA-SF and the subjective global assessment (SGA) were associated with postoperative complications [22].

Regarding the length of hospital stay, “at risk/malnourished” MNA-SF scores were significantly associated with a prolonged length of stay in our study. In a Chinese study comparing the MNA with the NRS 2002 in geriatric medical patients, both tools had a linear relationship with the length of stay, and agreement was found to be moderate between the two tools [23]. In a small sample of geriatric orthopaedic patients, those judged to be at risk of malnutrition or malnourished according to the MNA had a higher average length of stay compared to non-malnourished patients [24]. Similar results have been reported recently by Zhao et al. In this study, elderly non-cardiac surgical patients were assessed with the MNA-SF and the geriatric nutritional risk index (GNRI) with regard to postoperative delirium and length of stay. Both tools were independent predictors of prolonged LOS and performed equally in predicting longer hospitalisation [25]. These results highlight the need for preoperative malnutrition assessment in elderly patients, the optimisation of which before surgery can contribute to a shorter postoperative stay.

An important issue regarding malnutrition screening is the absence of a standardized global definition of malnutrition, a term that encompasses multiple nutritional disorders. The uncertainty is present not only amongst physicians but also amongst dieticians, [26] and although efforts have been made by ESPEN [27] and ASPEN [28] in the recent years, no global consensus has yet been reached. It is of paramount importance that a definition be given according to objective criteria, based on which screening tools will be validated in the future. Until a ‘gold standard’ tool or definition for the validation of screening tools is available and has gained wide acceptance worldwide, screening tools should be evaluated based on their association with postoperative outcomes, ideally through large-scale prospective studies comparing multiple tools.

Our study has limitations. It is a single-centre retrospective study, and therefore, a limited number of patients was studied with a consequent small number of postoperative outcomes, especially with regard to mortality. An inherent risk of bias due to the retrospective nature of the study is present, although careful prospective registration of postoperative complications was performed with low rates of missing data. Two validated screening tools were chosen for comparison, although other tools are also available for clinicians to choose from, such as the NRS 2002, the association of which with postoperative complications, mortality and length of stay was demonstrated in a meta-analysis by Sun et al. [29]. No gold standard method was used for the assessment of malnutrition, such as the SGA tool or assessment by a dietician, while another possible limitation is that our results can only be applied in the general surgical setting.

## 5. Conclusions

In the setting of preoperative malnutrition risk assessment in older patients undergoing operations of general surgery, the MNA-SF seems to be more consistently associated postoperative mortality and length of hospital stay compared with the MUST tool.

## Figures and Tables

**Table 1:** Baseline clinical characteristics of 302 elderly patients undergoing general surgery.

	*n* (%)
Female	130 (43)
Age (years)	
65–69	80 (27)
70–74	76 (25)
75–79	79 (26)
>79	67 (22)
MNA-SF	
Normal	223 (74)
At risk	56 (18)
Malnourished	23 (8)
MUST	
Low risk	194 (64)
Medium risk	67 (22)
High risk	41 (14)
Katz ADL categories	
Dependent (0–2)	22 (7)
Intermediate (3–4)	24 (8)
Independent (5–6)	255 (85)
Charlson’s index	
0	86 (29)
1–2	125 (41)
3–4	58 (19)
>4	31 (10)
Diagnosis of dementia	20 (7)
Diagnosis of cancer	122 (40)
POSSUM Operative Severity *	9 (8)
POSSUM Physical Status *	20 (8)
ASA class	
0–I	86 (29)
II	144 (48)
III–IV	60 (20)
Site of operation	
Hernia	69 (23)
Upper GI	14 (5)
HPB	33 (11)
Cholecystectomy	73 (24)
Lower GI	78 (26)
Soft tissue/thyroid/other	35 (12)
Postoperative complications	
Any complications	86 (29)
Serious complication	19 (6)
Death	5 (2)

* Median (interquartile), MUST: malnutrition universal screening tool, MNA-SF: Mini Nutritional Assessment–Short Form, ADL: activities of daily life, POSSUM: physiological and operative severity score for the enumeration of mortality, ASA: American Society of Anaesthetists, GI: Gastrointestinal, HPB: Hepato-pancreato-biliary Missing values < 3% for each variable.

**Table 2:** Univariate analysis of the association of MNA-SF and MUST tool with postoperative outcomes in 302 elderly patients undergoing operations of general surgery.

	Any Complication OR (95%)	*p* *	Serious Complications OR (95%)	*p* *	Postoperative Death OR (95%)	*p* *	Length of Stay (Days) Median (IQR)	*p*
MNA-SF								
Normal	Ref		Ref		Ref		4 (7)	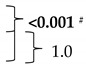
Atrisk	2.1 [1.2–4.0]	**0.014**	3.5 [1.3–9.5]	**0.011**	12.6 [1.3–123]	**0.029**	10 (14)	
Malnourished	1.6 [0.7–4.1]	0.287	1.0 [0.1–7.9]	0.975	10.1 [0.6–166]	0.106	9 (7)	
MUST								
Lowrisk	Ref		Ref		Ref		3 (6)	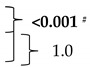
Mediumrisk	1.5 [0.8–2.8]	0.170	1.5 [0.5–4.1]	0.443	9.0 [0.9–88]	0.058	9 (12)	
Highrisk	2.2 [1.1–4.5]	**0.026**	0.5 [0.04–3]	0.358	4.8 [0.3–78]	0.269	9 (8)	
MNA-SF								
Normal	Ref		Ref		Ref		4 (7)	
At risk/malnourished	2.0 [1.1–3.4]	**0.02**	2.7 [1.1–7.0]	**0.030**	11.8 [1.1–108]	**0.018**	9.5 (11)	**<0.001** **^^^**
MUST								
Low risk	Ref		Ref		Ref		3 (6)	
Medium/High risk	1.8 [1.1–3.0]	**0.02**	1.1 [0.4–2.8]	0.919	7.4 [0.8–67]	0.057	9 (9)	**<0.001** **^^^**

MNA-SF: Mini Nutritional Assessment—Short Form, MUST: malnutrition universal screening tool, OR: odds ratio, IQR: interquartile range, serious complications are defined as Clavien–Dindo Grade > II. * Pearson’s Chi square. ^#^ Kruskal–Wallis test. ^ Mann–Whitney U test. Missing values < 4% for each variable. Values in bold are considered statistically significant.

**Table 3:** Multivariate analysis of the association of MNA-SF and MUST tool with postoperative outcomes in 302 elderly patients undergoing operations of general surgery.

	Any Complication AOR (95%CI)	*p* *	Serious Complications AOR (95%CI)	*p* *	Postoperative Death AOR (95%CI)	*p* *	Length of Stay (Days) AIR (95%CI)	*p* *
MNA-SF								
Normal	Ref	0.511	Ref	0.210	Ref	0.116	Ref	
Atrisk	1.5 [0.7–3.1]	0.252	2.6 [0.8–7.9]	0.104	16.9 [1.2–244]	**0.038**	1.5 [1.2–1.9]	**0.001**
Malnourished	1.3 [0.4–3.7]	0.649	0.7 [0.1–6.6]	0.766	5.3 [0.2–123]	0.304	1.1 [0.8–1.6]	0.599
MUST								
Lowrisk	Ref	0.700	Ref	0.213	Ref	0.462		
Mediumrisk	0.8 [0.4–2.8]	0.469	0.5 [0.1–2.0]	0.325	6.3 [0.3–127]	0.231	1.3 [1.0–1.6]	**0.048**
Highrisk	1.1 [0.4–2.6]	0.884	0.2 [0–1.4]	0.096	2.0 [0.1–50]	0.678	1.0 [0.8–1.4]	0.956
MNA-SF								
Normal	Ref		Ref		Ref		Ref	
At risk/malnourished	1.5 [0.8–2.8]	0.263	2.0 [0.7–5.8]	0.226	11.1 [0.9–131]	0.056	1.4 [1.1–1.7]	**0.004**
MUST								
Low risk	Ref		Ref		Ref		Ref	
Medium/High risk	0.9 [0.5–1.6]	0.641	0.4 [0.1–1.3]	0.114	3.7 [0.3–55]	0.346	1.2 [0.9–1.4]	0.120

MUST: Malnutrition Universal Screening Tool, MNA-SF: Mini Nutritional Assessment—Short Form. 95%CI: 95% confidence interval. AOR: adjusted odds ratio by multivariate logistic regression analysis. AIR: negative binomial regression adjusted incidence rate ratio. Multivariate analysis adjusted for ADL: activities of daily living, POSSUM operative severity: physiological and operative severity score for the numeration of mortality, POSSUM physiological score, Charlson comorbidity index. * Wald test. Values in bold are considered statistically significant.

## Data Availability

All data generated or analyzed during this study are included in this published article or are available from the corresponding author upon reasonable request.

## Supplementary Materials

The following are available online at https://www.mdpi.com/article/10.3390/jcm10245860/s1, Table S1: distribution of preoperative clinical variables between low and medium/high risk for malnutrition. Table S2: association of preoperative variables with postoperative outcomes in 302 older patients undergoing operations of general surgery. Table S3: association of site of the operation with postoperative outcomes in 302 older patients undergoing operation of general surgery. Table S4: distribution of malnutrition risk assessed by MUST and MNA-SF tools according to the site of operation in 302 older patients undergoing operations of general surgery. Table S5: univariate analysis of the association of MNA-SF and MUST tool with postoperative outcomes in 125 elderly patients undergoing upper/lower GI tract and hepatobiliary operations of general surgery. Table S6: multivariable analysis of predictors of any complications including MNA-SF categories. Table S7: multivariable analysis of predictors of serious complications including MNA-SF categories. Table S8: multivariable analysis of postoperative death including MNA-SF categories. Table S9: multivariable analysis of predictors of postoperative complications including MNA-SF normal vs. at risk/malnourished categories. Table S10: multivariable analysis of predictors of serious postoperative complications including MNA-SF normal vs. at risk/malnourished categories. Table S11: multivariable analysis of predictors of postoperative death including MNA-SF normal vs. at risk/malnourished categories. Table S12: multivariable analysis of predictors of any complication including MUST low vs. medium/high risk categories. Table S13: multivariable analysis of predictors of serious complications including MUST categories. Table S14: multivariable analysis of predictors of postoperative death including MUST categories. Table S15: multivariable analysis of predictors of any complication including MUST low vs. medium/high risk categories. Table S16: multivariable analysis of predictors of serious postoperative complications MUST low vs. medium/high risk categories. Table S17: multivariable analysis of postoperative death including MUST low vs. medium/high risk categories. Table S18: multivariable analysis of predictors of postoperative stay including MNA-SF categories. Table S19: multivariable analysis of predictors of postoperative stay including MNA-SF normal vs. at risk/malnourished categories. Table S20: multivariable analysis of predictors of postoperative stay including MUST categories. Table S21: multivariable analysis of predictors of postoperative stay including MUST low vs med/high risk categories.
