# Supplementary material for: A Comparison of the Malnutrition Universal Screening Tool (MUST) and the Mini Nutritional Assessment-Short Form (MNA-SF) Tool for Older Patients Undergoing General Surgery
Source: J Clin Med. 2021 Dec 14;10(24):5860. doi: 10.3390/jcm10245860 (PMC8704256; doi:10.3390/jcm10245860)
Supplement: Supplementary file 1 [file jcm-10-05860-s001.zip › jcm-1418185-supplementary.pdf]

SUPPLEMENT

JCM-1418185

"A comparison of the Malnutrition Universal Screening Tool (MUST) and the Mini Nutritional Assessment - Short Form (MNA-SF) tool for older patients undergoing general surgery."

Table S1. Distribution of preoperative clinical variables between low and medium/high risk for malnutrition.

	MNA risk			MUST risk		
	**Low N(%)**	**Med./high N(%)**	**P***	**Low N(%)**	**Med./high N(%)**	**P***
**Age group**						
65-69	65 (29.1)	15 (19)	0.08	56 (28.9)	24 (22.2)	0.07
70-74	56 (25.1)	20 (25.3)		47 (24.2)	29 (26.9)	
75-79	60 (26.9)	19 (24.1)		56 (28.9)	23 (21.3)	
>79	42 (18.8)	25 (31.6)		35 (18)	32 (29.6)	
**ASA class**						
I	66 (30.6)	19 (25.7)	0.263	62 (33)	23 (22.5)	**0.015**
II	111 (51.4)	33 (44.6)		97 (51.6)	47 (46.1)	
III	36 (16.7)	21 (28.4)		27 (14.4)	30 (29.4)	
IV	2 (0.9)	1 (1.4)		1 (0.5)	2 (2)	
**Charlson's index**						
0	77 (34.8)	9 (11.4)	**<0.01**	69 (25.8)	17 (15.9)	**<0.01**
1-2	99 (44.8)	26 (32.9)		84 (43.5)	41 (38.3)	
3-4	29 (13.1)	29 (36.7)		29 (15)	29 (27.1)	
>4	16 (7.2)	15 (19)		11 (5.7)	20 (18.7)	

ASA, American Society of Anaesthetists,

*Pearson Chi-Square

Table S2. Association of preoperative variables with postoperative outcomes in 302 older patients undergoing operations of General Surgery.

	**Any complication N (%)**	**P***	**Serious complications N (%)**	**P***	**Postoperative Death N (%)**	**P***	**Length of stay (days) Median(IQR)**	**P#**
**Age groups**								
65-69	20 (23.3)	0.83	7 (36.8)	0.301	1 (20)	0.192	3 (6)	0.051
70-74	23 (26.7)		4 (21.1)		1 (20)		6 (12)	
75-79	22 (25.6)		2 (10.5)		0 (0)		7 (9)	
>79	21 (24.4)		6 (31.6)		3 (60)		7 (8)	
**ADL categories**								
Dependent (0-2)	6 (7.1)	0.99	3 (15.8)	0.293	3 (60)	**<0.001**	7 (8)	0.402
Intermediate (3-4)	7 (8.2)		2 (10.5)		0 (0)		4 (9)	
Independent (5-6)	72 (84.7)		14 (73.7)		2 (40)		6 (8)	
**Charlson's index**								
0	13 (15.3)	**0.005**	3 (15.8)	0.428	1 (20)	0.897	3 (2.25)	**<0.001**
1-2	39 (45.9)		10 (52.6)		2 (40)		6 (8)	
3-4	24 (28.2)		5 (26.3)		1 (20)		10 (7)	
>4	9 (10.6)		1 (5.3)		1 (20)		9 (12.75)	
**ASA class**								
0-I	24 (29.3)	0.24	7 (36.8)	0.24	0 (0)	0.067	4 (7)	**0.011**
II	36 (43.9)		6 (31.6)		2 (40)		4.5 (8)	
III-IV	22 (26.8)		6 (31.6)		3 (60)		9 (12)	

*Pearson Chi-Square

#Median Test

*Median (interquartile). MUST: malnutrition universal screening tool. MNA-SF: Mini Nutritional Assessment-Short Form. ADL: activities of daily life. POSSUM: Physiological and Operative Severity Score for the enUmeration of Mortality. ASA: American Society of Anaesthetists.

-Missing values <3% for each variable.

Table S3. Association of site of the operation with postoperative outcomes in 302 older patients undergoing operation of General Surgery.

	**Total patients N (%)**	**Any complication N (%)**	**P***	**Serious complications N (%)**	**P***	**Postoperative Death N (%)**	**P***	**Length of stay (days) Median(IQR)**	**P#**
Hernia	69 (23)	11 (12.8)	**<0.01**	3 (15.8)	**0.035**	0 (0)	0.274	4 (3)	**<0.001**
Upper GI	14 (5)	4 (4.7)		0 (0)		0 (0)		19 (19.5)	
HPB	33 (11)	20 (23.3)		6 (31.6)		2 (40)		25 (22)	
Cholecystectomy	73 (24)	13 (15.1)		4 (21.1)		1 (20)		5 (5.5)	
Lower GI	78 (26)	32 (37.2)		6 (31.6)		2 (40)		11 (7)	
Soft tissue/ thyroid/other	35 (12)	6 (7)		0 (0)		0 (0)		6 (9.25)	

*Pearson Chi-Square
#Median Test

Table S4. Distribution of malnutrition risk assessed by MUST and MNA-SF tools according to the site of operation in 302 older patients undergoing operations of General Surgery.

	**MUST low risk N (%)**	**MUST medium/high risk N (%)**	**P***	**MNA low risk N (%)**	**MNA medium/high risk N (%)**	**P***
Hernia	59 (30.4)	10 (9.3)	**<0.01**	66 (29.6)	3 (3.8)	**<0.01**
Upper GI	2 (1)	12 (11.1)		6 (2.7)	8 (10.1)	
HPB	15 (7.7)	18 (16.7)		16 (7.2)	17 (21.5)	
Cholecystectomy	59 (30.4)	14 (13)		60 (26.9)	13 (16.5)	
Lower GI	33 (17)	45 (41.7)		46 (20.6)	32 (40.5)	
Soft tissue/ thyroid/other	26 (13.4)	9 (8.3)		29 (13)	6 (7.6)	

*Pearson Chi-Square

Table S5. Univariate analysis of the association of MNA-SF and MUST tool with postoperative outcomes in 125 elderly patients undergoing upper/lower GI tract and hepatobiliary operations of general surgery.

	Any complication OR [95%]	p*	Serious complications OR [95%]	p*	Postoperative Death OR [95%]	p*	Length of stay (days) Median(IQR)	p
**MNA-SF**								
Normal	Ref		Ref		Ref		10(7)	0.566
At risk	1.0 [0.5-2.3]	0.929	1.5[0.4-5.2]	0.543	3.5 [0.3-40]	0.310	10(15)	
Malnourished	1.1 [0.4-3.3]	0.827	0.6[0.07-5.7]	0.695	8.4 [0.7-98]	0.09	10(9)	
**MUST**								
Low risk	Ref		Ref		Ref		10(11)	0.526
Medium risk	0.8 [0.3-1.8)	0.555	0.9 [0.2-3.2]	0.892	8.3 [0.4-165]	0.165	10(14)	
High risk	1.2 [0.5-2.9]	0.722	0.2 [0.03-2.2]	0.214	5.1 [0.2-130]	0.321	10(8)	
**MNA-SF**								
Normal	Ref		Ref		Ref		10(7)	
At risk/malnourished	1.1 [0.5-2.2]	0.867	1.2[0.4-4]	0.748	3.7[0.4-36.8]	0.261	10(13)	0.892^
**MUST**								
Low risk	Ref		Ref		Ref		10(11)	
Medium/High risk	1.1 [0.5-2.2]	0.825	1.2[0.7-2.2]	0.541	6.6 [0.3-120]	0.149	10(10)	0.569^

Table S6. Multivariable analysis of predictors of any complications including MNA-SF categories.

	P	Odds Ratio	95% C.I. for Odds Ratio	
			Lower	Upper
**MNA-SF**				
Normal	0.511	Ref		
At risk	0.252	1.518	0.743	3.105
Malnourished	0.649	1.283	0.440	3.741
**Activities of daily life score (ADL)**	0.861	1.045	0.635	1.721
**Charlson's index**	0.794	1.048	0.739	1.484
**POSSUM Operative Severity***	**0.012**	1.089	1.019	1.164
**POSSUM Physical Status***	**0.030**	1.052	1.005	1.102

95%C.I., 95% confidence interval of the Odds Ratio

Table S7. Multivariable analysis of predictors of serious complications including MNA-SF categories.

	P	Odds Ratio	95% C.I. for Odds Ratio	
			Lower	Upper
**MNA-SF**				
Normal	0.210	Ref		
At risk	0.104	2.556	0.825	7.919
Malnourished	0.766	0.714	0.078	6.566
**Activities of daily life score (ADL)**	0.123	0.575	0.284	1.162
**Charlson's index**	0.339	0.739	0.398	1.374
**POSSUM Operative Severity***	0.093	1.087	0.986	1.198

**POSSUM Physical Status***	0.179	1.053	0.976	1.136

Table S8. Multivariable analysis of postoperative death including MNA-SF categories.

	P	Odds Ratio	95% C.I. for Odds Ratio	
			Lower	Upper
**MNA-SF**				
Normal	0.116	Ref		
At risk	**0.038**	16.881	1.168	244.077
Malnourished	0.304	5.245	0.223	123.619
**Activities of daily life score (ADL)**	**0.009**	0.152	0.037	0.622
**Charlson's index**	0.638	0.756	0.236	2.421
**POSSUM Operative Severity***	0.147	1.118	0.962	1.300
**POSSUM Physical Status***	0.119	1.124	.0970	1.302

Table S9. Multivariable analysis of predictors of postoperative complications including MNA-SF normal vs. at risk/malnourished categories. .

	P	Odds Ratio	95% C.I. for Odds Ratio	
			Lower	Upper
**MNA-SF**				
Normal		Ref		
At risk/malnourished	0.263	1.454	0.755	2.800
**Activities of daily life score (ADL)**	0.815	1.060	0.649	1.731
**Charlson's index**	0.805	1.045	0.738	1.479
**POSSUM Operative Severity***	**0.012**	1.089	1.019	1.164
**POSSUM Physical Status***	**0.029**	1.053	1.005	1.102

Table S10. Multivariable analysis of predictors of serious postoperative complications including MNA-SF normal vs. at risk/malnourished categories.

	P	Odds Ratio	95% C.I. for Odds Ratio	
			Lower	Upper
**MNA-SF**				
Normal		Ref		
At risk/malnourished	0.226	1.962	0.659	5.839
**Activities of daily life score (ADL)**	0.197	0.639	0.323	1.261
**Charlson's index**	0.304	0.724	0.392	1.340
**POSSUM Operative Severity***	0.081	1.091	0.989	1.204
**POSSUM Physical Status***	0.163	1.055	0.978	1.138

Table S11. Multivariable analysis of predictors of postoperative death including MNA-SF normal vs. at risk/malnourished categories.

	P	Odds Ratio	95% C.I. for Odds Ratio	
			Lower	Upper
**MNA-SF**				
Normal				
At risk/malnourished	0.056	11.083	0.937	131.091
**Activities of daily life score (ADL)**	**0.008**	0.191	0.056	0.653
**Charlson's index**	0.516	0.685	0.219	2.147
**POSSUM Operative Severity***	0.138	1.125	0.963	1.315
**POSSUM Physical Status***	0.105	1.130	0.975	1.310

Table S12. Multivariable analysis of predictors of any complication including MUST low vs. medium/high risk categories.

	P	Odds Ratio	95% C.I. for Odds Ratio	
			Lower	Upper
**MUST**				
Low risk	0.700	Ref		
Medium risk	0.469	0.762	0.365	1.591
High risk	0.884	1.069	0.440	2.597
**Activities of daily life score (ADL)**	0.836	1.054	0.640	1.735
**Charlson's index**	0.680	1.075	0.761	1.519
**POSSUM Operative Severity***	**0.005**	1.105	1.030	1.186
**POSSUM Physical Status***	**0.030**	1.053	1.005	1.103

Table S13. Multivariable analysis of predictors of serious complications including MUST categories..

	P	Odds Ratio	95% C.I. for Odds Ratio	
			Lower	Upper
**MUST**				
Low risk	0.213	Ref		
Medium risk	0.325	0.509	0.132	1.957
High risk	0.096	0.151	0.016	1.394
**Activities of daily life score (ADL)**	0.096	0.542	0.263	1.116
**Charlson's index**	0.618	0.855	0.462	1.582
**POSSUM Operative Severity***	**0.012**	1.145	1.030	1.271
**POSSUM Physical Status***	0.229	1.047	0.971	1.129

Table S14. Multivariable analysis of predictors of postoperative death including MUST categories.

	P	Odds Ratio	95% C.I. for Odds Ratio	
			Lower	Upper
**MUST**				
Low risk	0.462	Ref		
Medium risk	0.231	6.284	0.311	127.116
High risk	0.678	1.986	0.078	50.494
**Activities of daily life score (ADL)**	**0.008**	0.193	0.057	0.656
**Charlson's index**	0.913	0.938	0.299	2.948
**POSSUM Operative Severity***	0.683	1.042	0.854	1.272
**POSSUM Physical Status***	0.236	1.099	0.940	1.284

Table S15. Multivariable analysis of predictors of any complication including MUST low vs. medium/high risk categories.

	P	Odds Ratio	95% C.I. for Odds Ratio	
			Lower	Upper
**MUST**				
Low risk		Ref		
Medium/High risk	0.641	0.856	0.445	1.646
**Activities of daily life score (ADL)**	0.924	1.024	0.625	1.678
**Charlson's index**	0.623	1.090	0.774	1.535
**POSSUM Operative Severity***	**0.005**	1.107	1.031	1.187
**POSSUM Physical Status***	**0.035**	1.050	1.003	1.100

Table S16. Multivariable analysis of predictors of serious postoperative complications MUST low vs. medium/high risk categories.

	P	Odds Ratio	95% C.I. for Odds Ratio	
			Lower	Upper
**MUST**				
Low risk		Ref		
Medium/High risk	0.114	0.356	0.099	1.280
**Activities of daily life score (ADL)**	0.109	0.563	0.279	1.137
**Charlson's index**	0.503	0.812	0.441	1.494
**POSSUM Operative Severity***	**0.009**	1.150	1.036	1.277
**POSSUM Physical Status***	0.170	1.054	0.978	1.135

Table S17. Multivariable analysis of postoperative death including MUST low vs. medium/high risk categories.

	P	Odds Ratio	95% C.I. for Odds Ratio	
			Lower	Upper

**MUST**				
Low risk				
Medium/High risk	0.346	3.684	0.245	55.505
**Activities of daily life score (ADL)**	**0.008**	0.226	0.074	0.683
**Charlson's index**	0.708	0.813	0.274	2.408
**POSSUM Operative Severity***	0.446	1.073	0.895	1.287
**POSSUM Physical Status***	0.155	1.116	0.959	1.298

Table S18. Multivariable analysis of predictors of postoperative stay including MNA-SF categories.

		**Adjusted Incidence Rate**	**95% Wald Confidence Interval for Adjusted Incidence Rate**	
	**P**		Lower	Upper
**MNA-SF**				
Normal	.	1	.	.
At risk	**0.001**	1.480	1.165	1.880
Malnourished	0.599	1.101	0.768	1.579
**Charlson's index**				
0	.	1	.	.
1-2	**0.014**	1.336	1.061	1.682
3-4	**<0.001**	1.892	1.426	2.511
>4	0.107	1.365	0.935	1.992
**Katz ADL categories**				
Independent (5-6)	.	1	.	.
Intermediate (3-4)	0.299	1.283	0.801	2.055
Dependent (0-2)	0.263	1.234	0.854	1.783
**POSSUM Operative Severity***	**<0.001**	1.090	1.064	1.116
**POSSUM Physical Status***	**0.015**	1.020	1.004	1.036

Table S19. Multivariable analysis of predictors of postoperative stay including MNA-SF normal vs. at risk/malnourished categories. .

		**Adjusted Incidence Rate**	**95% Wald Confidence Interval for Adjusted Incidence Rate**	
	**P**		Lower	Upper
**MNA-SF**				
Normal	.	1	.	.
At risk/malnourished	**0.004**	1.380	1.109	1.717

**Charlson's index**				
0	**.**	1	.	.
1-2	**0.011**	1.348	1.070	1.698
3-4	**<0.001**	1.863	1.404	2.473
>4	0.090	1.387	0.950	2.026
**Katz ADL categories**				
Independent (5-6)	.	1	.	.
Intermediate (3-4)	0.269	1.305	0.814	2.094
Dependent (0-2)	0.208	1.267	0.876	1.832
**POSSUM Operative Severity***	**<0.001**	1.089	1.064	1.115
**POSSUM Physical Status***	**0.012**	1.020	1.004	1.036

Table S20. Multivariable analysis of predictors of postoperative stay including MUST categories.

		Adjusted Incidence Rate	95% Wald Confidence Interval for Adjusted Incidence Rate	
	**P**		Lower	Upper
**MUST**				
Low risk	.	1	.	.
Medium risk	0.048	1.259	1.002	1.580
High risk	0.956	1.008	0.749	1.358
**Charlson's index**				
0	.	1	.	.
1-2	**0.009**	1.362	1.079	1.719
3-4	**<0.001**	2.020	1.528	2.671
>4	0.055	1.451	0.991	2.123
**Katz ADL categories**				
Independent (5-6)	.	1	.	.
Intermediate (3-4)	0.258	1.317	0.817	2.122
Dependent (0-2)	0.394	1.180	0.807	1.724
**POSSUM Operative Severity***	**<0.001**	1.091	1.064	1.118
**POSSUM Physical Status***	**0.034**	1.017	1.001	1.033

Table S21. Multivariable analysis of predictors of postoperative stay including MUST low vs med/high risk categories.

		Adjusted Incidence Rate	95% Wald Confidence Interval for Adjusted Incidence Rate	
	**P**		Lower	Upper
**MUST**				
Low risk	.	1	.	.
Medium/high risk	0.120	1.176	0.958	1.444
**Charlson's index**				
0	.	1	.	.
1-2	**0.011**	1.351	1.070	1.705
3-4	**<0.001**	2.025	1.531	2.679
>4	0.058	1.447	0.988	2.120
**Katz ADL categories**				
Independent (5-6)	.	1	.	.
Intermediate (3-4)	0.225	1.344	0.834	2.168
Dependent (0-2)	0.268	1.236	0.849	1.799
**POSSUM Operative Severity***	**<0.001**	1.090	1.063	1.117
**POSSUM Physical Status***	**0.027**	1.018	1.002	1.034
